# Novel Drugs Obtained via Biotransformation—In Memory of the Late Scientists Frieder Schauer and Peter Grunwald

**DOI:** 10.3390/microorganisms11071734

**Published:** 2023-07-01

**Authors:** Mariam Gaid, Annett Mikolasch

**Affiliations:** Institute of Microbiology, University of Greifswald, Felix-Hausdorff-Str. 8, 17489 Greifswald, Germany

The development of novel drugs is a complex process that requires cost-effective and sustainable techniques. Microbial enzymes, either isolated or natively integrated within whole microbial cells, are effective eco-friendly tools that can be used as catalysts for the green synthesis of new bioactive agents [1].

Unlike conventional chemical synthesis, enzyme-mediated catalysis works under eco-friendly conditions and can successfully target regio- and stereo-specific reactions. In these reactions, macromolecules can be engineered and repurposed to have a function that is new to nature. In the pharmaceutical industry, the implementation of biocatalysis is needed and this approach should continue being investigated to enrich drug development and enhance new pharmacological values [2]. This approach has also been integrated to support the biodegradation of complex materials or to target the selective biosynthesis of special compounds that can be used as educts in medical or dental applications [3,4]. The selection of microbial strains or biocatalysts is based on the protein sequences of the target enzymes. Genomics and transcriptomics have led to significant advancements, providing researchers with innovative genetic tools to identify target enzymes or microorganisms [5].

This Special Issue includes six original research articles. These articles clearly demonstrate the power of microbial biotransformation with an update on proven enzyme-mediated routes, using single-step biotransformation or enzyme cascade, which can offer advantages over traditional chemical synthesis [6].

Mikolasch and Hahn studied the antibacterial efficacy and cytotoxicity of new bioactive heterodimers and heterotrimers with sulfonamide or sulfone structures. The tested compounds were biosynthesized using the oxidative coupling function of the extracellular enzyme, laccase C, from *Trametes* spec. [7]. Similarly, laccases from three different microbial sources (*Pycnoporus cinnabarinus*, *Myceliophthora thermophila* and *Trametes* spec.) were used to couple 2,5-dihydroxy-N-(-2-hyroxyethyl)-benzamide with kanamycin, tobramycin and gentamicin to form new aminoglycoside antibiotics. Interestingly, the new products showed no cytotoxicity and comparable or better antibacterial activities than the parent structures [8]. Meene and colleagues [9] identified bacterial isolates obtained from a brackish water site of Lake Balkhash (Kazakhstan) as *Pseudomonas veronii* and *Paenibacillus apiarius*. The authors tested the bacteriolysis ability of these isolates to selected Gram-negative (*Pseudomonas putida* SBUG 24 and *Escherichia coli* SBUG 13) and Gram-positive strains (*Micrococcus luteus* SBUG 16 and *Arthrobacter citreus* SBUG 321). The analysis of the bacteriolysis process revealed the identification of a potential antibiotic compound from *Paenibacillus apiarius*. In another study, promising candidates of arachidonic acid derivatives that have pharmacological and therapeutic values were obtained using the regioselective catalytic property of an unspecific peroxygenase from the ascomycetous fungus *Truncatella angustata* (*Tan*UPO). The structural simulation of the *Tan*UPO protein could explain the selective oxyfunctionalization of arachidonic acid substrates [10]. The extracellular cutinase (ACut2)-containing supernatant from a non-conventional yeast, *Blastobotrys* (*Arxula*) *raffinosifermentans* (*adeninivorans*), was introduced as a new biocatalyst for the production of monoesters. It was studied to catalyze the desymmetrization of dicarboxylic acid diester diethyl adipate to monoester monoethyl adipate as a main product after optimizing the reaction conditions, showing a promising yield after up-scaling [11]. Ali and colleagues [12] extended the findings of previous research and presented a potential candidate for the digestion of agro-residual substrates. The wood fungus *Staphylotrichum longicolleum* FW57 was tested for its chitinase activity to digest sugarcane bagasse and maize leaves. It is noteworthy that the catalytic efficiency of the obtained FW57 enzyme mixture shows that it could be used to replace available commercial enzyme cocktails.

In summary, the articles presented in this Special Issue show the significance of microbial enzymes and strains in various biocatalytic processes and applications. Their findings can contribute to the development of industrialized cost-effective microbial transformation technologies.
